# Draft Genome Sequence of *Pantoea ananatis* Strain 1.38, a Bacterium Isolated from the Rhizosphere of *Oryza sativa* var. Puntal That Shows Biotechnological Potential as an Inoculant

**DOI:** 10.1128/genomeA.01547-17

**Published:** 2018-01-25

**Authors:** Esaú Megías, Fábio Bueno dos Reis Junior, Renan Augusto Ribeiro, Francisco Javier Ollero, Manuel Megías, Mariangela Hungria

**Affiliations:** a Facultad de Biología, Departamento de Microbiología, Universidad de Sevilla, Seville, Spain; b Embrapa Cerrados, Soil Microbiology, Planaltina, Federal District, Brazil; c CNPq, Brasília, Federal District, Brazil; d Embrapa Soja, Soil Biotechnology, CP 231, Londrina, Paraná, Brazil

## Abstract

*Pantoea ananatis* 1.38 is a strain isolated from the rhizosphere of irrigated rice in southern Spain. Its genome was estimated at 4,869,281 bp, with 4,644 coding sequences (CDSs). The genome encompasses several CDSs related to plant growth promotion, such as that for siderophore metabolism, and virulence genes characteristic of pathogenic *Pantoea* spp. are absent.

## GENOME ANNOUNCEMENT

The genus *Pantoea* encompasses plant-pathogenic, commensal, and endophytic species, and the beneficial species may promote plant growth (1–3). Our research group isolated and characterized several beneficial *Pantoea* strains from rice (*Oryza sativa* L.) paddies of the Guadalquivir River marshes in southern Spain (4–6), and we have sequenced the genome of *Pantoea ananatis* strain 1.38, isolated from the rhizosphere of the variety Puntal. The bacterium has phosphate solubilization activity, siderophore and auxin production, and cellulose, lipase, and pectinase activities. In experiments performed under greenhouse conditions, strain 1.38 increased the biomasses of rice (5 to 8%), maize (*Zea mays* L.) (15 to 17%), and, when coinoculated with *Rhizobium tropici*, common bean (*Phaseolus vulgaris* L.) (35 to 40%).

DNA extraction and sequencing were performed as previously described (4–6). Paired-end reads obtained by shotgun sequencing on the MiSeq platform allowed a genome coverage of 230-fold. The FASTQ files were assembled by the A5-miseq pipeline (*de novo* assembly) (7). The genome was estimated at 4,869,281 bp, assembled in 23 contigs, with a G+C content of 53.3 mol%; there are two plasmids of about 180 and 60 Mb. The average nucleotide identity (ANI) values of the strain 1.38 genome with the whole genomes of *P. ananatis* strains LMG 2665^T^, AMG 501, and AMG521 were 96.41, 96.37, and 99.16%, respectively. Therefore, strains AMG521 and 1.38 might comprise a specific group of *P. ananatis* strains from the marshes of the Guadalquivir River.

Sequences were submitted to the Rapid Annotations using Subsystems Technology (RAST) server (8), and 4,644 DNA coding sequences (CDSs) were identified, with 58% classified in 530 subsystems. Several genes are related to stress response (166 CDSs, 3.5% of the genome), including those for response to osmotic and oxidative stress and to cold and heat shock, and those for choline, betaine, and trehalose biosynthesis. There are several transcriptional regulators, with at least 23 belonging to the LysR family.

Protein secretion systems (types I to VIII) can play important roles in the interaction of *P. ananatis* with plants. Strain 1.38 carries genes of types II, IV (pilus type IV, INC conjugal transfer, and conjugal transfer proteins TraI, TraB, TraC, TraK, and TraW), VI, and VII (fimbrial type I) secretion systems, which are slightly different from those of strain AMG521, as the type V is absent in strain 1.38. There are no virulence genes typical of plant-pathogenic strains of *P. ananatis* (9), such as the YhcA protein of the type IV secretion system, phage P2 (GpU), or phage P7 (Gp4); in addition, strain 1.38 carries chaperones of the PapD family, which are typical of beneficial *P. ananatis* strains.

Strain 1.38 has genes involved in siderophore metabolism (siderophore aerobactin in outer membrane, rhizobactin, *IutA* receptor, and the *iucA*, *iucB*, and *iucD* genes) and a system of recognition and transport of siderophores (FhuA, FhuD, FhuB, and FhuC). There are also quorum-sensing genes (*N*-acyl-l-homoserine lactose synthetase, *N*-acyl-l-homoserine lactose hydrolase, *N*-3-oxohexanoyl-l-homoserine lactose, and *N*-3-oxooctanoyl-l-homoserine lactose) similar to those of the AMG 501 and AMG521 strains (4, 6).

### Accession number(s).

The whole-genome shotgun project has been deposited at DDBJ/EMBL/GenBank under the accession number NKXT00000000 (SUBID SUB2841407, BioProject number PRJNA393047, BioSample number SAMN07313992). The version described in this paper is version NKXT01000000.
